# Bioinspired DNA Origami Quasi-Yagi Helical Antenna with Beam Direction and Beamwidth Switching Capability

**DOI:** 10.1038/s41598-019-50893-8

**Published:** 2019-10-04

**Authors:** Syed Imran Hussain Shah, Sungjoon Lim

**Affiliations:** School of Electrical and Electronics Engineering, Chung-Ang University, Seoul, Korea

**Keywords:** Electrical and electronic engineering, Mechanical engineering

## Abstract

We propose a bioinspired origami quasi-Yagi helical antenna with beam direction and beamwidth switching capability based on transformable DNA origami structure. Each DNA molecule consists of a double helical chain, and its length can be transformed by folding and unfolding. When three transformable origami DNA structures are applied to the quasi-Yagi helical antenna, beam direction and beamwidth can be controlled by folding and unfolding the origami DNA. The transformable DNA structures act as driven, director and reflector elements. The proposed DNA origami antenna provides four beam direction switching states (three states with narrow beamwidth and one state with wide beamwidth) at fixed frequency of 1.9 GHz. For example, the main beam direction of the proposed antenna can be steered to −30°, 0°, +30° and −40° for states 1, 2, 3 and 4, respectively. State 4 provides a 3-dB wider beamwidth of 104°, whereas the beamwidth of other states is narrower than 64°. The proposed concept is numerically and experimentally demonstrated.

## Introduction

The term origami refers to folding and unfolding technology where two-dimensional sheets are creased and then folded to realize three-dimensional (3D) compact and deformable structures. Origami has recently gained much attention in several research fields, with origami-based systems exhibiting many interesting features including, but not limited to, programmable curvature^1^, tunable stiffness^2^, self-foldability^3^, programmable collapse^4^ and multistability^2,5,6^. These features make origami a promising approach for flexible electronics^7^, self-foldable robots^8^, soft pneumatic actuators^9^, etc. Complex architectural models can also be designed by employing origami technology. An origami heart stent has been presented for medical applications^10^, and an origami foldable telescope has been put forth for space exploration^11^. Origami-oriented technology can be a good alternative to design flexible, deployable and low-cost antennas compared with conventional antenna fabrication techniques. Various antenna geometries can be easily achieved using origami technology that are not possible by conventional fabrication. However, only a few origami antennas have been reported, and they have limitations of instability and non-robustness.

A pattern reconfigurable antenna can change the radiation direction for a fixed operating frequency by adjusting its aperture. Pattern reconfigurable antennas significantly improve system performance because they can avoid noise sources and electronic jamming, simultaneously improving system security and saving energy by providing better signal directivity towards the desired users. Modern wireless communication technologies have strong requirements for pattern reconfigurable antennas for satellite, radar and military communication^12,13^. Consequently, various techniques have been proposed for pattern reconfiguration, including mechanical and electrical approaches^14^. Pattern reconfiguration can also be achieved by origami antennas, where the pattern is reconfigured by folding and unfolding the antenna, altering the geometry. For example, in^15^, a pattern reconfigurable origami antenna was presented where the omni-directional pattern could be reconfigured to a directional pattern by folding the antenna. In^16^, a foldable microstrip antenna was proposed using origami concept. The antenna could be transformed from a monopole with an omni-directional pattern to a microstrip patch type with a directional pattern. A mode reconfigurable antenna using Nojima origami concept was presented in^17^. The antenna could be operated in directional mode in the folded state and switched to omni-directional mode in the unfolded state. However, the operating frequency also changed during folding and unfolding.

Table 1 shows origami antennas fabricated from readily available paper sheets^15,17–22^, which makes them unstable for extensive folding and sometimes repeatedly folding-unfolding. These antennas were not robust and were unsuitable for outdoor antenna applications. Their dielectric properties, including the dielectric constant and loss tangent, were also influenced and altered by environmental factors, including humidity and rain^23^. In^16,24^, antennas using shape memory polymers (SMPs) were presented, which could convert the structure in the pre-programmed geometry under certain stimuli. Although SMP-based origami antennas are more robust than antennas fabricated from paper, SMP fabrication is time consuming, requires significant laboratory facilities and has limited adherence to conductors. Therefore, the current study proposes a robust and flexible polyethylene terephthalate (PET) film-based origami antenna, overcoming the various limitations of previous origami antennas. The proposed antenna is inspired by the DNA of living cells.

**Table 1:** Previous origami antennas and proposed reconfigurable antenna.

Ref.	Antenna type	Substrate material	Operating frequency (GHz)	Pattern reconfigurable	Number of modes	Switchable beamwidth	Efficiency	Peak gain (dBi)	Robust
^15^	Dipole	Paper	0.48 & 2.5	Yes	2	No	N/A	4.5	No
^16^	Patch	SMPs	2.2	Yes	2	No	N/A	4.5	Yes
^17^	Helical	Paper	0.6 &1.6	Yes	2	No	N/A	4.18	No
^18^	Yagi	Paper	3	No	1	No	43.5–73%	9.5	No
^19^	Monopole array	Paper	3.5	No	1	No	57%	5	No
^20^	Yagi-loop array	Paper	1.31	No	1	No	N/A	10.4	No
^24^	Patch array	SMPs	2.3	No	2	No	N/A	N/A	Yes
^21^	Helical	Paper	0.86–2.14	No	1	No	N/A	6.79	No
^22^	Yagi	Paper	1.9	No	1	No	64%	7.3	No
This work	Yagi-helical	PET	1.85	Yes	4	Yes	85–96%	8	Yes

**Figure 1:**
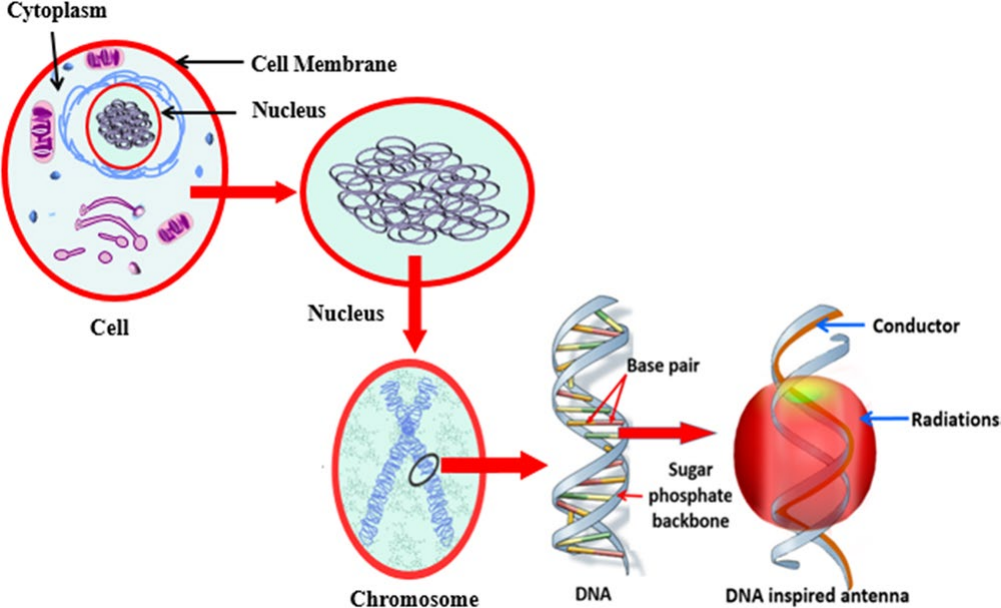
DNA folding and unfolding features, motivation for pattern reconfigurable origami antennas.

A living cell can be considered an elementary life unit. The cell body consists of four biomolecules: deoxyribonucleic acid (DNA), lipids, polysaccharides and proteins. The cellular genome comprises chromosomes where DNA is held in base pairs. Figure 1 demonstrates DNA evolution from the cell. DNA is a commonly known molecule that contains minute genetic instructions and biological information required for the growth, development and reproduction of living creatures. Crick and Watson (1953) discovered DNA at the Cavendish Laboratory and showed that the DNA geometry consists of double helical chains coiled around a common axis (Fig. 1). The double helical structure consists of a sugar and phosphate backbone with four bases: thymine, adenine, cytosine and guanine. DNA’s structure and folding and unfolding capability are interesting features that can be employed to design frequency and pattern reconfigurable antennas with efficient folding and compact packaging.

Therefore, this paper proposes a robust bioinspired origami quasi-Yagi helical antenna based on transformable DNA origami structure. Beam direction and beamwidth can be switched by exploiting origami DNA folding and unfolding features, with three origami DNA structures operating as driven, director and reflector elements. The proposed DNA origami antenna provides four beam direction switching states (three with narrow beamwidth and one with wide beamwidth) at fixed frequency of 1.9 GHz. For example, the main beam direction of the proposed antenna can be steered to −30°, 0°, +30° and −40° for states 1, 2, 3 and 4, respectively, with state 4 providing a 3-dB wider beamwidth of 104°, whereas the beamwidth of other states is narrower than 64°.

The proposed antenna has the novelty of the reconfigurable helix configuration compared to conventional helix antennas. The novel reconfigurable helix configuration is applied to the beam direction switching capability of the quasi-Yagi antenna. The proposed antenna is the first beam scanning Yagi antenna using the reconfigurable helix.

## Results

### Antenna design and numerical simulation

Most previously reported origami antennas were not robust and inappropriate for outdoor applications due to being fabricated from paper substrate (see Table 1). Therefore, we propose a DNA-inspired origami antenna constructed from robust PET sheets to overcome the identified limitations of previous origami antennas and ensure suitability for outdoor applications. The PET film was 0.175 mm thick, with dielectric constant and loss tangent = 3.0 and 0.002, respectively^25^. PET substrate is a promising candidate for origami antennas because of its lower loss tangent, flexibility and robustness to repeated folding and unfolding cycles.

**Figure 2:**
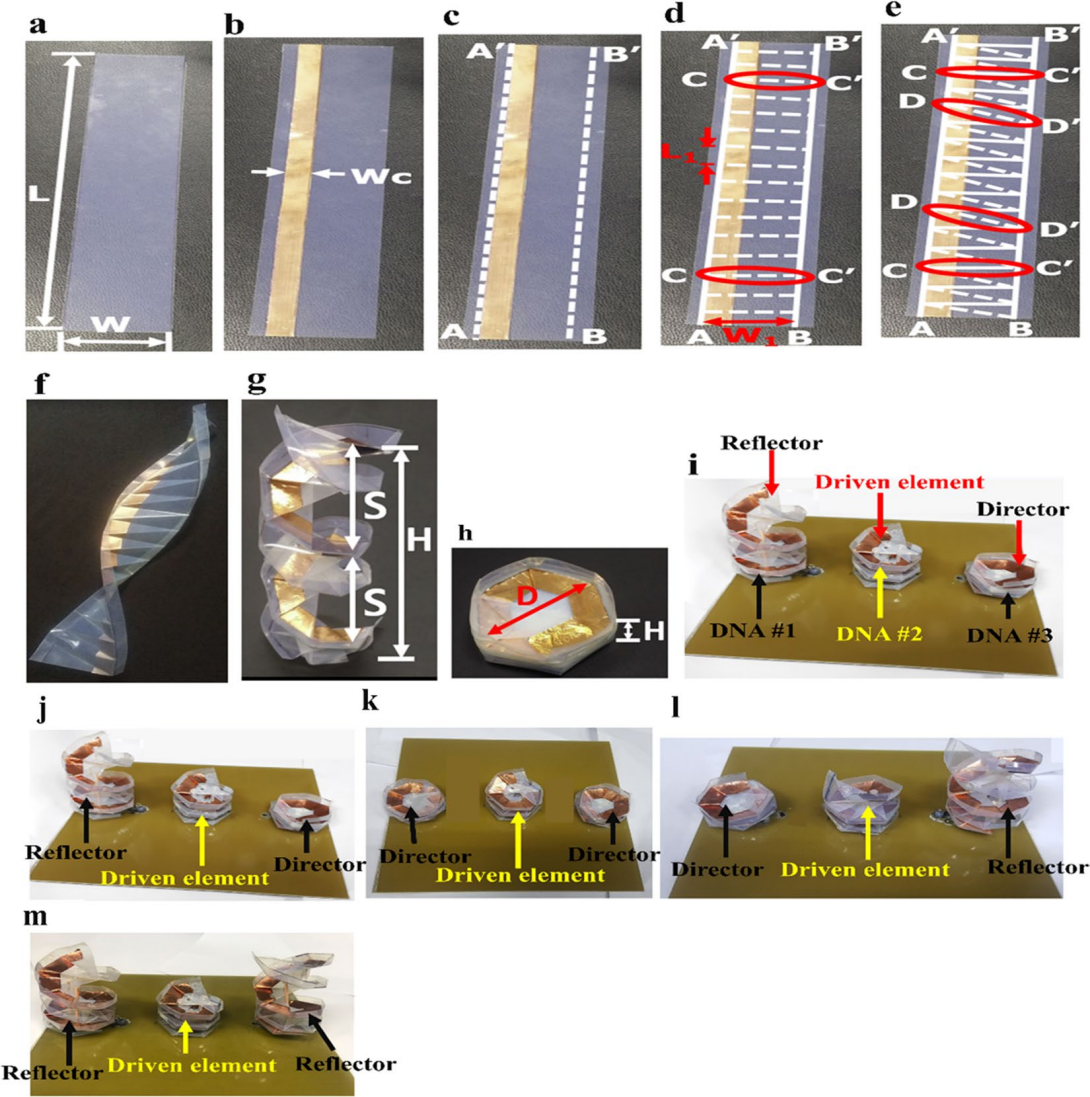
Proposed antenna fabrication. Step 1: (**a**) rectangular sheet of PET substrate with *L* = 300 mm and *W* = 53 mm; (**b**) PET sheet with attached conductor pattern; Step 2: (**c**) fold and unfold along AAʹ and BBʹ; (**d**) fold and unfold along CCʹ, *L*_1_ = 19 mm; Step 3: (**e**) fold and unfold along DDʹ; (**f**) sheet after rectangular and triangular creases. Origami DNA in (**g**) unfolded form, (h) folded form. (**i**) Proposed origami DNA antenna in (**j**) state 1, (**k**) state 2, (**l**) state 3 and (**m**) state 4.

Figure 2 shows the fabrication process for the origami DNA antenna. A rectangular PET film sheet (300 × 53 mm) was obtained (Fig. 2(a)), and a copper film (width *W*_*C*_ = 12 mm and thickness = 0.1 mm) was added to the PET sheet to provide the conductor pattern (Fig. 2(b)). For convenience, the PET sheet was marked with segments AAʹ and BBʹ (Fig. 2(c)). Creases along AAʹ and BBʹ were introduced by folding and unfolding the sheet along these lines to provide the backbone of the DNA.

1. The sheet was folded and unfolded along all horizontal segments denoted by CCʹ to create creases along these lines and divided the sheet into 16 rectangular segments (Fig. 2(d)) with length *L*_1_ = 19 mm and width *W*_1_ = 41 mm.
2. The sheet was folded and unfolded along the diagonal lines DDʹ to create creases that divided each rectangular section into two symmetrical triangular segments (Fig. 2(e)). Figure 2(f) shows the final PET sheet after making all the creases by folding and unfolding.

The folded sheet can then be twisted from the top to realize the proposed origami DNA, which can be repeatedly folded and unfolded, as shown in Fig. 2(g,h), respectively. Let S be the spacing between turns of the origami DNA. In the folded state, S can be reduced to 1 mm, whereas in the unfolded state, S maximum = 100 mm. However, for this study, we utilized optimized S = 25 for the unfolded DNA. The same fabrication procedure was repeated to fabricate three similar origami DNA geometries, called DNA1, DNA2 and DNA3 for convenience. DNA2 was used as the driven element for the proposed antenna, which was excited by co-axial feeding.

The proposed antenna was simulated and analyzed using the ANSYS high-frequency structure simulator (HFSS). We set the dielectric constant and loss tangent of the PET film to 3.0 and 0.002, respectively, and the copper film was assumed to have conductivity of 4.4 × 10^5^ S/m^26^. Other dielectric properties of FR4 were assumed as ε_r_ = 4.4 and tan δ = 0.02.

### Operating mechanism

#### Reconfiguration by switching reflector and director

We used three origami DNAs (DNA1, DNA2 and DNA3), where DNA2 was the driven element and DNA1 and DNA3 were the parasitic elements of the proposed quasi-Yagi helical antenna. Spacing between turns of the driven element could be varied to tune the frequency. We selected spacing S = 15 mm to operate the antenna in the range of 1.8 GHz to 1.9 GHz. The spacing between turns of the parasitic elements (DNA1 and DNA3) was optimized to provide improved directivity. When spacing between turns of either DNA1 or DNA3 = 25 mm, the parasitic element operated as reflector in the unfolded state, and when spacing was reduced to 1 mm, the parasitic element worked as a director in the folded state. The projected electrical length was reduced in the folded state, so DNA1 and DNA3 acted as directors. The effective electrical length was increased in the unfolded state so that DNA1 and DNA3 acted as reflectors. Thus, the antenna could operate in four different states by simply folding and unfolding the parasitic elements. Hence, the tuning of this spacing replicated the switching mechanism between the reflector and director, as shown in Fig. 2(j–m). Therefore, we obtained the four different states.

#### Beam direction and beamwidth switching capability

**Figure 3:**
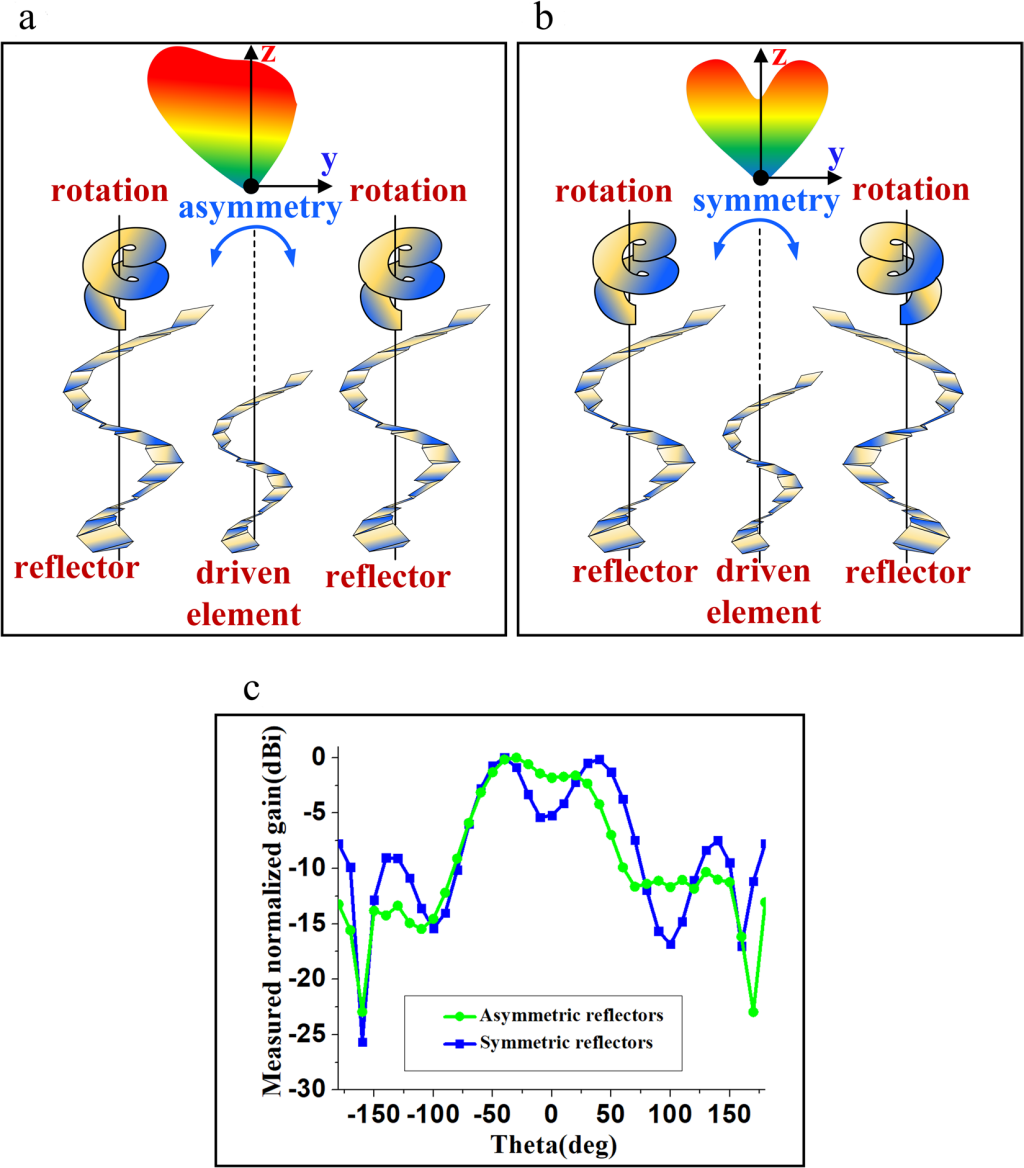
Possible configurations of two reflectors for state 4: (**a**) asymmetric configuration, (**b**) symmetric configuration and (**c**) radiation patterns.

In state 1, DNA1 operated as the director and DNA3 operated as the reflector. For this state, the main beam of the antenna was inclined towards the director and the opposite side of the reflector at a radiating angle of 30°. In state 2, we set the spacing between DNA1 and DNA3 at 1 mm and both origami DNAs were working as directors. The main beam was directed towards 0**°**. In state 3, we set S = 1 and 25 for DNA1 and DNA3, respectively. The beam direction for the third state was directed towards −30**°**. In state 4, the radiation pattern was dependent on the orientation of the two reflectors. Figure 3(a,b) illustrate the asymmetric and symmetric orientation of the two reflectors, respectively. Figure 3(c) shows the simulated radiation patterns of the proposed antenna in state 4 when S = 25 for both DNA1 and DNA3 and the two reflectors are asymmetric and symmetric. As shown in Fig. 3(a,c), the maximum radiation occurs at −40° with 100° 3-dB beamwidth when two reflectors are asymmetric. On the other hand, when the two reflectors are symmetric as shown in Fig. 3(b,c), the beam bifurcates into the −40° and +40° maximum radiation directions with a narrower 3-dB beamwidth of 40°. In this work, we designed the asymmetric orientation for two reflectors because of high peak gain at the other states.

**Figure 4:**
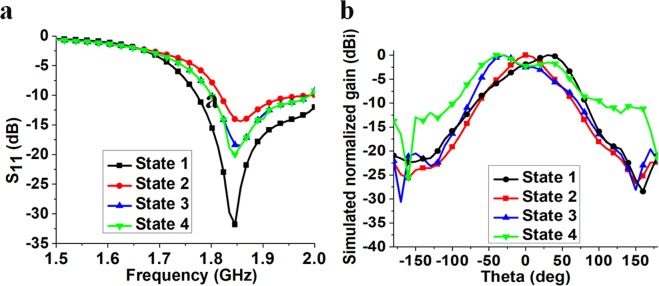
Simulated (**a**) reflection coefficients and (**b**) normalized gain for the proposed antenna in the four states at 1.9 GHz.

Figure 4(a) shows the simulated S-parameters of all four states. The resonant frequency is 1.85, 1.85, 1.84 and 1.84 GHz in states 1, 2, 3 and 4, respectively. The common 10-dB impedance bandwidth for all four states is 1.81–1.97 GHz. Therefore, good impedance matching is exhibited for all states. In addition, Fig. 4(b) shows the simulated normalized gain for the four states. As discussed, the maximum beam direction is 30°, 0°, −30° and −40° in state 1, 2, 3 and 4, respectively. In addition, the 3-dB beamwidth is 61°, 56°, 63° and 100° in state 1, 2, 3 and 4, respectively.

### Measurement results

The spacing between the turns of the driven element (DNA2) was fixed at 15 mm using spacers to obtain operating frequency of the antenna in the range of 1.8 GHz to 1.9 GHz. The parasitic elements (DNA1 and DNA3) were folded and unfolded to act as directors or reflectors. Thus, the antenna can operate in four different states.

#### S-parameter measurements

**Figure 5:**
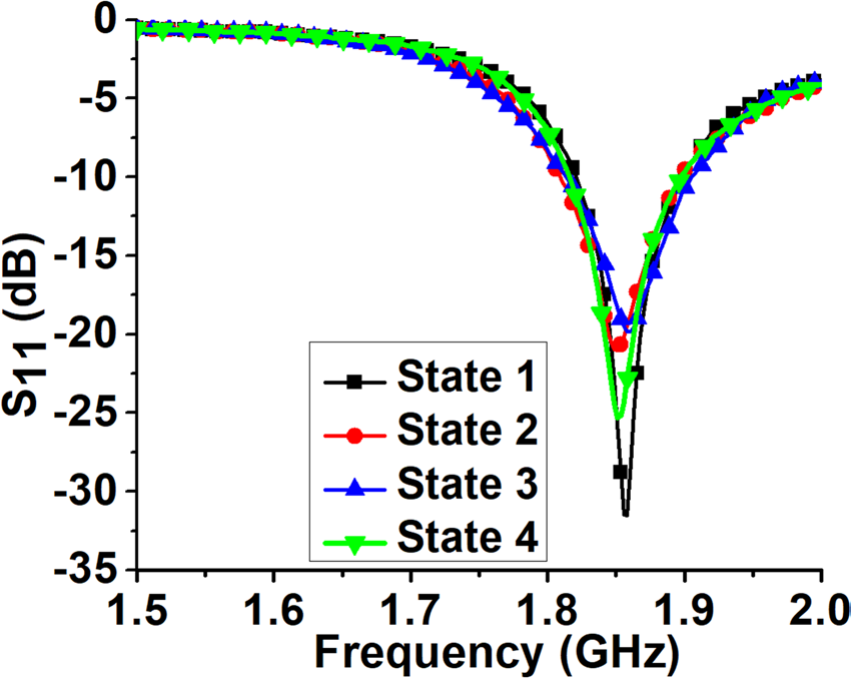
Measured (S_11_) for the proposed pattern reconfigurable antenna in the four states.

Firstly, the reflection coefficients for the four states were measured using an Anritsu vector network analyzer (MS2038C). Figure 5 shows the measured reflection coefficients (S_11_) for the proposed pattern reconfigurable antenna in the four states. The resonant frequency was 1.85 GHz in all states. The 10-dB impedance bandwidth covered 1.82–1.9 GHz, and the resonant frequency and impedance bandwidths were completely matched for all the states, which is required to obtain different patterns at a fixed frequency. Most of the beam steering antennas using conventional tuning techniques suffer from issues related to the reduction of common impedance bandwidth. This is because of the notable variation in 10-dB impedance bandwidth during different beam steering states^27^. On the contrary, the proposed geometry appeared to be a much better alternative to avoid this limitation, providing consistently large common impedance bandwidth for all the cases.

#### Far-field measurements

**Figure 6:**
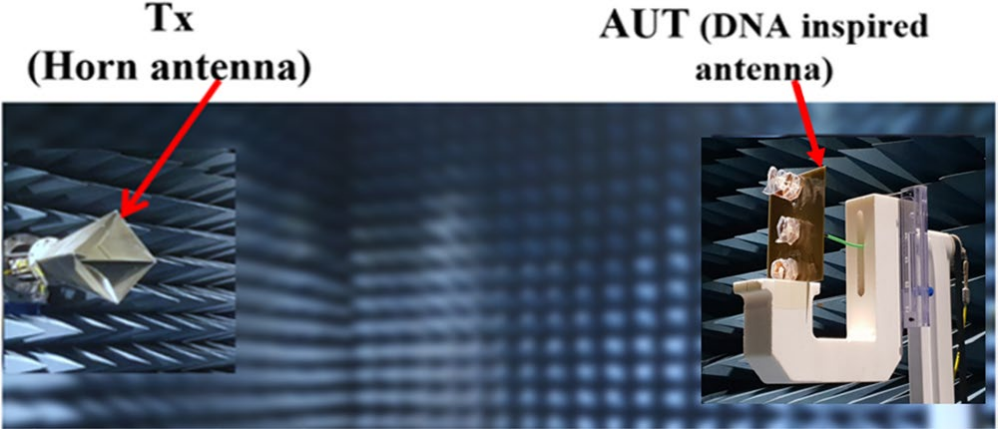
Measurement setup to obtain 3D radiation pattern in the anechoic chamber.

**Figure 7:**
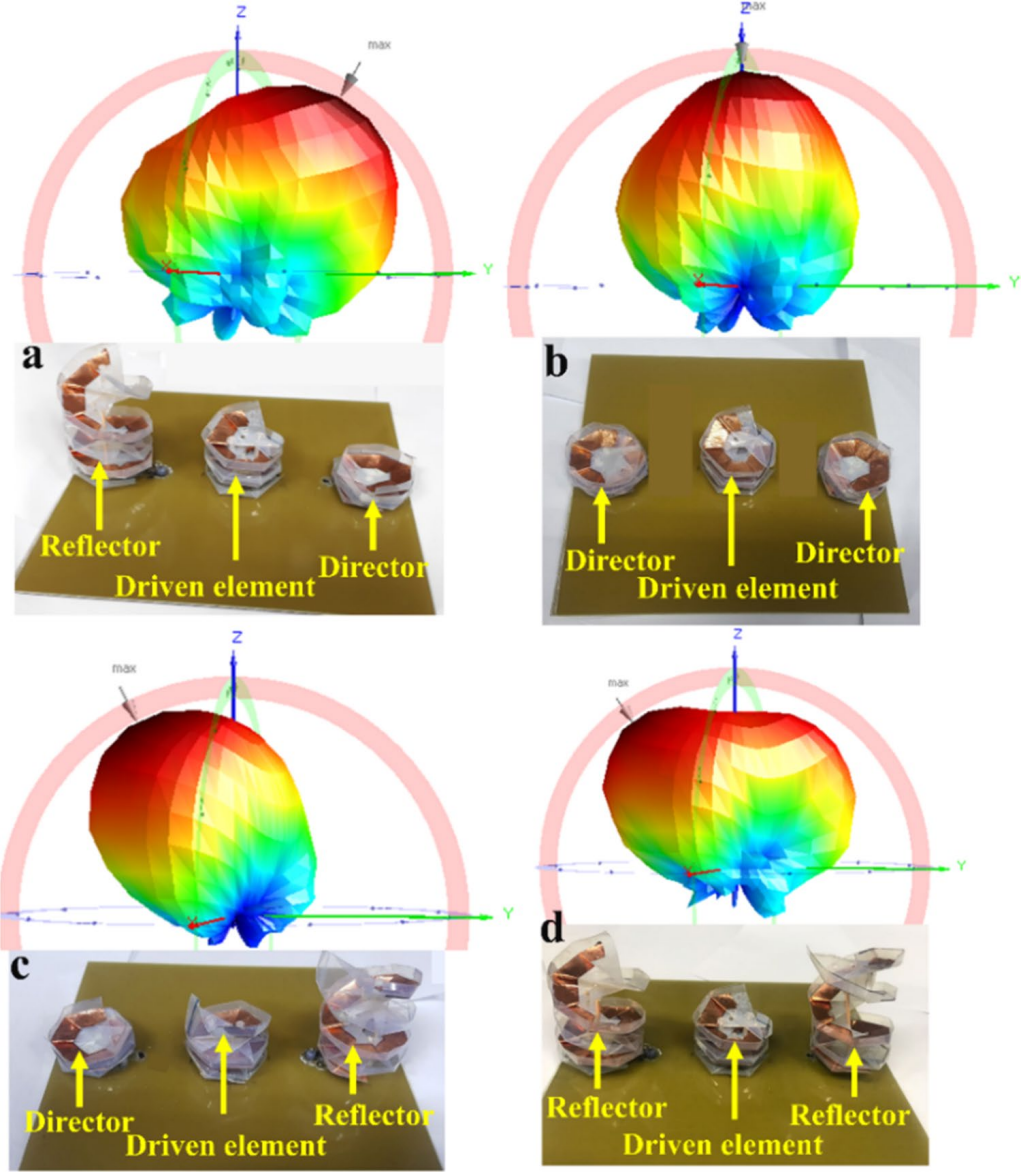
Measured 3D radiation pattern for the proposed antenna in (**a**) state 1, (**b**) state 2, (**c**) state 3 and (**d**) state 4.

The radiation pattern of the fabricated antenna was measured for all four operating states in the commercial ORBIT/FR far-field measurement setup in the shielded radio frequency anechoic chamber. Figure 6 shows the measurement setup to obtain the radiation pattern for the proposed origami DNA antenna in an anechoic chamber, with the resulting 3D radiation patterns shown in Fig. 7. It is observed that the maximum beam direction occurs at 30°, 0°, −30° and −40° in state 1, 2, 3 and 4, respectively.

**Figure 8:**
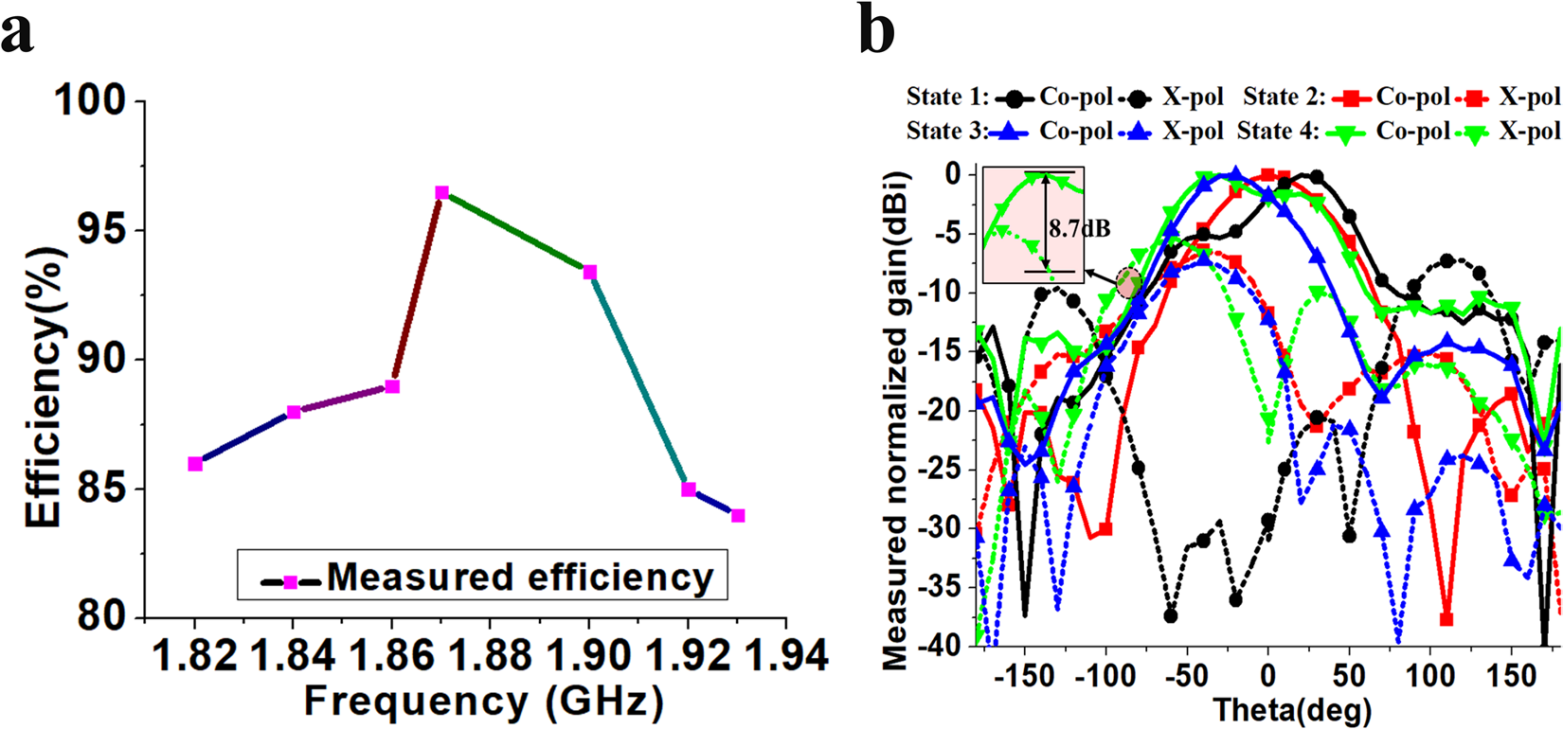
Measured (**a**) radiation efficiency and (**b**) normalized radiation pattern for the proposed antenna at 1.9 GHz.

Figure 8(a) shows the measured radiation efficiency of the fabricated antenna from 1.82 to 1.93 GHz in state 1. The variation of measured efficiency of the antenna was obtained from 85–96% across the operating frequency range. Thus, a significant improvement in radiation efficiency was achieved as compared to the previously reported origami antennas (see Table 1). The low-loss, robust and flexible PET film was essential to increase the proposed DNA-inspired antenna’s efficiency.

Figure 8(b) shows the measured normalized radiation pattern of the fabricated antenna at 1.9 GHz. The main beam was directed towards 30**°** for state 1. Due to the shorter electrical length in the folded state, DNA3 acted as director, while DNA1 acted as reflector because of its longer electrical length in the unfolded state. Peak gain for this state 1 is obtained as 6.54 dBi with 3-dB beamwidth = 63**°**. Both parasitic elements (DNA1 and DNA3) were folded for state 2 and acted as directors. Hence, the main beam was directed towards 0**°**. Peak gain in this state = 7.38 dBi and 3-dB beamwidth = 56**°**. State 3 is the opposite of state 1, with DNA1 acting as director due to its folded state and DNA3 acting as reflector due to its unfolded state. Hence, the main beam was directed towards −30°. Peak gain in this state was 8.06 dBi and 3-dB beamwidth = 62**°**. A discrepancy between the measured peak gain for Modes I and III was observed due to the folding imperfections using the manual process. Both parasitic elements acted as reflectors in state 4, providing peak gain = 6.6 dBi and the main beam was directed towards −40**°**. State 4 exhibited 3-dB beamwidth = 104**°**, from −52**°** to 52**°**. For clarity, the radiation patterns of various states are summarized in Table 2.

**Table 2:** Beam direction and beamwidth switching response for the proposed origami antenna.

Antenna state	Function of DNA1	Function of DNA2	Function of DNA3	Beam direction	3-dB beamwidth	Peak gain (dBi)
1	Reflector	Driven Element	Director	30°	63°	6.54
2	Director	Driven Element	Director	0°	56°	7.38
3	Director	Driven Element	Director	−30°	62°	8.06
4	Reflector	Driven Element	Reflector	−40°	104°	6.6

The cross-polarization levels are plotted with the co-polarization level in Fig. 8(b). For states 1, 2, 3 and 4, the difference of the co- and cross-polarization levels is 21, 12, 9 and 8.7 dB, respectively, in the main beam direction of each state.

## Conclusions

We propose a robust bioinspired origami quasi-Yagi helical antenna, with a structure inspired by the transformable nature of living tissue DNA. The proposed antenna beam direction and beamwidth switching capabilities were demonstrated using three transformable origami DNA structures.

The proposed DNA origami antenna provided four switchable states to control beam direction and beamwidth at fixed frequency of 1.9 GHz. The main beam directions of the narrow beams could be steered to −30°, 0° and +30° for states 1, 2 and 3, respectively. State 4 provided a wider 3-dB beamwidth = 104°, whereas the other three states had 3-dB beamwidth ≤63°.

The proposed antenna is low cost due to realization using PET film, and the fabrication process is simple and fast. This is the first robust antenna from the origami approach. The proposed antenna is suitable for military applications, where the beam direction switching capability would provide secure communication, and it would also be favorable for providing different wireless communication services in urban and rural environments. Although there is a limitation of low switching speed associated with the origami technology. Nevertheless, switching speed can be increased using actuators, which will be demonstrated in our future research outcomes.

### Numerical simulations

Before going to fabrication, the proposed antenna is designed and simulated using the ANSYS High Frequency Structure Simulator (HFSS). A radiation boundary is used to emulate free space by truncating the infinite free space to a finite calculation domain. The radiation box is taken λ/4 (or λ/2) away from each side of the antenna. The electrical conductivity of copper was considered as 4.4 × 10^5^ S/m^26^. The dielectric properties of the PET substrate were taken as relative permittivity (ε_r_ = 3.0) and tangential loss (tan δ = 0.002)^25^, while the dielectric properties of FR4 were considered as ε_r_ = 4.4 and tan δ = 0.02. The antenna was excited by using a wave port.

### Bioinspired origami antenna fabrication

To experimentally demonstrate the bioinspired DNA-based antenna for the beam direction and beamwidth switching concept, we fabricated three origami DNA geometries from robust and flexible PET sheets. The step-by-step fabrication procedure of an origami DNA geometry is presented in Fig. 2. A copper film was attached to provide the antenna conductor pattern. DNA2 was used as the driven element with DNA1 and DNA3 as the parasitic elements. A conductor-based FR4 substrate was utilized for the ground plane. The spacing between the turns of the driven element (DNA2) was fixed using 15-mm spacers to obtain operating frequency of the antenna in the range of 1.8 GHz to 1.9 GHz. The parasitic elements (DNA1 and DNA3) were folded and unfolded to act as directors or reflectors. The projected electrical length was reduced in the folded state, so DNA1 and DNA3 acted as directors. The effective electrical length was increased in the unfolded state so that DNA1 and DNA3 acted as reflectors. Thus, the antenna could operate in four different states.

### Microwave measurement

The measurements were carried out in two steps. First, the reflection coefficients for the four states were measured using an Anritsu vector network analyzer (MS2038C) while folding and unfolding the parasitic elements, as shown in Fig. 5. The network analyzer was calibrated using a calibration kit. The antenna was connected to the network analyser by an SMA connector of the driven element. The radiation pattern of the fabricated antenna was measured for all four operating states in the commercial ORBIT/FR far-field measurement setup in the shielded radio frequency anechoic chamber, as shown in Fig. 6. The data for the 3D radiation pattern were collected by rotating the fabricated prototype of the antenna.
